# DIGITAL APPROACHES TO ROUTINE COGNITIVE SCREENING FOR OLDER ADULTS IN PRIMARY CARE SETTINGS

**DOI:** 10.1093/geroni/igae098.2211

**Published:** 2024-12-31

**Authors:** Louisa Thompson, Molly Lawrence, Charles Eaton

**Affiliations:** Brown University Medical School, Lincoln, Rhode Island, United States; Brown University, Providence, Rhode Island, United States; Brown University Medical School, Providence, Rhode Island, United States

## Abstract

Cognitive screening remains under-utilized for older adults in primary care due to time constraints and limitations of existing measures. Well-validated digital assessments could potentially increase screening efficiency and accuracy. We compared two new digital cognitive tools with the Montreal Cognitive Assessment (MoCA) in a sample of 32 dementia-free older adults (ages 55-85) completing annual follow-up visits with a primary care provider (PCP). Participants self-administered the Boston Online Cognitive Assessment (BOCA), an online measure with alternate forms, twice prior to (1-4 weeks before and day of) an upcoming PCP follow-up visit. At their visit, they completed the Digital Clock and Recall (Linus Health DCRTM), a 5-minute, provider-administered tablet-based measure. Finally, they completed the MoCA with a research coordinator or behavioral health staff at the clinic. Five PCPs aided in protocol development and participated in data collection. The sample is currently 54% female and 81% White. Test-retest reliability for the BOCA was excellent (r =.81). BOCA score (time 1) was correlated with scores for the DCR and MoCA at the p <.05 level. The association between the DCR and MoCA approached significance. On an exit survey, 79% of participants said that they would prefer to do cognitive screening at home before their appointment, compared to in the clinic (21%). These preliminary data replicate excellent test re-test reliability for the BOCA and demonstrate good convergent validity between the BOCA and two provider-administered screening measures. Next, we will compare test accuracies to detect impairment and their associations with demographics variables.

